# Suboptimal cardiovascular risk factor identification and management in patients with rheumatoid arthritis: a cohort analysis

**DOI:** 10.1186/ar4118

**Published:** 2012-12-13

**Authors:** Shailey S Desai, James D Myles, Mariana J Kaplan

**Affiliations:** 1 Division of Rheumatology, University of Michigan Health System, 1500 East Medical Center Dr., Ann Arbor, MI 48109, USA; 2 Michigan Institute for Clinical and Health Research, University of Michigan Medical School, 2800 Plymouth Road, Building 400, Ann Arbor, MI 48109, USA

## Abstract

**Introduction:**

Accelerated cardiovascular (CV) disease significantly contributes to increased mortality in rheumatoid arthritis (RA) patients, with a risk comparable to the one observed in patients with type 2 diabetes mellitus (DM). Part of this enhanced risk in RA is attributed to traditional cardiovascular risk factors (CRFs). The aims of this study were to determine how often traditional CRFs are identified and managed by (a) rheumatologists, compared with primary care physicians (PCPs) in RA patients; and (b) PCPs among patients with RA, DM, and the general population (GP).

**Methods:**

A retrospective cohort study compared age/gender/ethnicity-matched patients from three groups: RA, DM, and GP (without RA or DM); *n = *251 patients per group. Electronic patient records were reviewed during a continuous 12-month period between June 2007 and April 2011 to assess whether CRFs were identified and managed.

**Results:**

In RA patients, PCPs managed obesity, BP, and lipids significantly more often than did rheumatologists. PCPs managed obesity, BP, and lipids significantly more often in diabetic patients than in the other two groups, and more often in the GP than in RA patients. In patients with elevated BMI, PCPs managed weight in 68% of the DM group, 46% of the GP, and 31% of the RA group (*P *< 0.0001 for all groups; *P *= 0.006 between RA and GP groups).

**Conclusions:**

Rheumatologists identify and manage CRFs less frequently than PCPs. PCPs manage CRFs less frequently in RA patients, compared to the GP and DM. Given the increased CV risk associated with RA, physicians need to more aggressively manage CRFs in these patients.

## Introduction

In patients with rheumatoid arthritis (RA), the leading cause of mortality is accelerated atherosclerotic cardiovascular disease (CVD), with standardized mortality ratios between 1.3 and 3 [1,2]. Patients with RA have a twofold increased risk for myocardial infarction (MI), and a 10-year risk of CV events that is 60% higher than that in the general population [3,4]. The increased CV risk in RA patients is considered secondary to both disease-specific mechanisms associated with enhanced inflammatory burden, and to traditional CV risk factors (CRFs), such as diabetes mellitus (DM)/insulin resistance, smoking, obesity, hypertension, and dyslipidemia [5-8].

RA and DM share a similarly increased risk of CV events [9,10]. However, although the frequency and severity of preclinical atherosclerosis is equal in RA and DM of similar duration, a differential impact seems to exist when comparing traditional risk factors versus systemic inflammation in both diseases. Traditional risk factors seem to play a greater role in CVD associated with DM, whereas systemic inflammation appears to play a greater role in RA [11]. Preliminary guidelines exist for CV risk prevention in RA, but these are certainly far more clearly established and validated in DM [12,13].

We hypothesized that traditional CRFs are not as frequently managed in patients with RA, as compared with patients with DM or with the general population (GP). We based this assumption on several observations. First, the awareness of the increased CV risk and primary prevention guidelines differs between RA and DM, as supported by the discrepancy in the strength of CV risk-prevention guidelines [12,13]. Second, several studies indicate potential suboptimal identification and treatment of CRFs in RA [14,15]. Third, as in DM, symptoms of angina and MI often go unrecognized in RA. Patients with RA are twice as likely to develop silent MIs and sudden cardiac death than is the general population [16]. Finally, it has been shown that patients with chronic illnesses have unrelated conditions that are often undertreated [17].

This study analyzed how frequently traditional CRFs were identified and managed by rheumatologists compared with PCPs in patients with RA in a tertiary care center. In addition, this study assessed how frequently these traditional CRFs were identified and managed by PCPs among patients with RA, DM, and the GP.

## Materials and methods

### Study design

We performed a retrospective cohort study to compare identification and management of traditional CRFs at a tertiary care center among age-, gender-, and ethnicity-matched patients from three groups: RA, type 2 DM, and GP (without RA or DM). Specifically, we determined how frequently CRFs were identified and managed by (a) rheumatologists and primary care physicians (PCPs) in patients with RA; and (b) PCPs in patients with RA, compared with those with DM or with the GP. Electronic patient records were reviewed during a continuous 12-month period, at some time between June 2007 and April 2012, to assess whether CRFs were identified and managed. Identification was defined as documenting the CRF at any visit. Management was defined as documenting a plan to address the CRF.

### Patient population

Patients from all three groups were required to have established care at the University of Michigan Health System (UMHS) for at least a 12-month period. Lists of patients were obtained from the University of Michigan Medical Center Information Technology (MCIT) after IRB approval. No patient consent was required, given that the study was a retrospective chart review. For the RA group, adult patients were included if they had an ICD9 diagnosis code of 714.0 (RA), had no diagnosis of type 1 or type 2 diabetes, and had been evaluated by a rheumatologist and a PCP in the UMHS during the same 12-month period. Once this list was obtained, the records were reviewed, and only those patients who fulfilled the 1987 ACR criteria for RA, with 1 or more years of disease duration, were included [18]. Patients with a diagnosis of systemic lupus erythematosus, undifferentiated connective tissue disease, Still's disease, other well-defined connective tissue diseases, polyarthritis due to a viral illness, or an uncertain diagnosis of RA were excluded. For the DM group, patients were included if they had an ICD9 diagnosis code of 250.00-250.92, consistent with type 2 diabetes. Once this list was obtained, the records were reviewed, and only those who fulfilled a diagnosis of type 2 diabetes, per the accepted criteria, were included [19]. Patients with a diagnosis of glucose intolerance who did not fulfill criteria for DM were excluded. For the GP group, patients were excluded if they had a diagnosis of RA and/or DM. Patients were age, race, and ethnicity matched among the three groups via random selection.

### Data collection and analysis

#### Baseline characteristics

For the RA group, the 12-month period for review was determined by finding the most recent visit to the Rheumatology Outpatient Clinic with a match of at least one PCP visit during the preceding 12 months. For the other two groups, this period was defined as the 12 months preceding the most recent PCP visit. Given that all rheumatology and primary care physician visit records were available in the same electronic medical record system and that all providers had access to this system, it is considered that the rheumatologists and PCPs monitoring a given RA patient were aware that the patient was under the care of both departments in the health system.

Demographic and clinical characteristics at baseline were assessed and captured. These included age, race, gender, smoking status, duration of disease, medications, and C-reactive protein (CRP) level. Smoking status was defined as a lifelong nonsmoker, past smoker, or current smoker. Duration of disease was determined by how long the symptoms leading to diagnosis had been ongoing, or when the diagnosis was made, if symptomatic duration was not mentioned in the records. Notable classes of medications that patients were taking included disease-modifying antirheumatic agents (DMARDs), biologics, insulin, antiglycemic agents, NSAIDs, antihypertensives, lipid-lowering agents, anticoagulation, and aspirin. The type and dosage for each class of medication was recorded. For patients with RA, the most recent CRP level was recorded, if available within 2 years of the visit. For patients with diabetes, the most recent hemoglobin A1c was recorded, if available within 2 years of the visit. For every patient, a personal history of hypertension (HTN), hyperlipidemia (HL), coronary artery disease (CAD), myocardial infarction (MI), and cerebrovascular accident (CVA) was noted if present. A family history of CAD, MI, or CVA was noted and recorded as positive if present in any first-degree relative, including parents, siblings, or children. The age at onset of a particular CV event was not considered in the definition of a family history, as the majority of physician notes did not include this information.

#### CRF assessment

Two components composed each CRF: identification and management. The CRF interventions included smoking cessation, weight-management strategies, BP control, lipid profile control, and fasting blood glucose (FBG) management strategies.

Smoking cessation was considered as identified if a physician assessed and recorded a patient's smoking status, regardless of the actual status. The issue was considered to be managed if a physician either encouraged continued smoking abstinence for a lifelong nonsmoker or former smoker, or if the physician provided active smoking-cessation counseling or medical treatment for a current smoker.

Exercise status was defined as whether or not a patient exercised. If a patient was found to exercise, the frequency of exercise was determined as infrequent (1 to 2 times weekly or less), moderate (3 to 4 times weekly), or regular (5 or more times weekly). If a patient was found to exercise, but no frequency was recorded, then it was assumed that the patient exercised regularly. If no mention of exercise was made, then this section was listed as no data.

Weight was considered to be identified if a physician noted exercise status, any recent weight changes, or dietary habits. Simply recording a weight in the physical examination was not sufficient to consider as weight identification. Weight was considered to be managed if the physician encouraged exercise initiation or continuation, or dietary interventions, when warranted.

BPs were determined as an average of measurements taken over the 12-month period, calculated separately for systolic and diastolic values, along with the number of measurements taken for each patient. If more than 10 measurements were taken over a given year, then only the most recent 10 measurements were included. BP was recorded as identified if a physician recorded a measurement for any visit. BP was recorded as managed if a physician recorded that abnormal BPs needed to be further monitored, if BP counseling was provided, or if antihypertensive drugs were prescribed or adjusted.

Cholesterol-profile monitoring was split into three categories: high-density lipoprotein (HDL), low-density lipoprotein (LDL), and triglyceride measurements. Each category was considered to be identified if any of the following was true: (a) a cholesterol measurement within the prior 3 years was documented in the clinic note; (b) a lipid profile was obtained after documentation of a plan to monitor it; or (c) documentation *alone *existed of a plan to obtain a lipid profile at the patient's most recent clinic visit within the 12-month study period. Each cholesterol category was considered to be managed if documentation of any of the following was found: (a) the need to recheck an abnormal cholesterol level; (b) discussion of lifestyle modifications; or (c) prescription or adjustment of cholesterol-lowering agents.

FBG was considered to be identified and managed in a similar manner as cholesterol. Of note, the blood glucose level was recorded only if known to be a fasting level.

### Statistical analysis

Kruskal-Wallis test or Wilcoxon Rank Sum test was used for continuous and χ^2 ^test for categoric outcomes without accounting for matching. A subgroup analysis was performed to assess the frequency in which patients with abnormal values for a specific CRF had it identified and managed. Abnormal values were considered as follows: BMI > 25, active smokers, SBP > 120, DBP > 80, HDL < 40, triglycerides > 150, and FBG > 100. Regarding LDL, an abnormal value was determined for each patient based on the calculated Framingham LDL goal, with a separate abnormal subgroup analysis performed based on this value [20]. Another subgroup analysis was performed, excluding all patients with a personal history of CAD, MI, and/or CVA, to determine the frequency of CRF identification and management among these patients. All data are presented as mean ± standard deviation or count and percentage.

## Results

### Baseline characteristics

In total, 359 patients were initially identified as having RA; this number decreased to 251 patients once the records were reviewed and noneligible patients were excluded, as described earlier in Methods. The study included a total of 753 patients, with 251 patients in each group (Table 1). The mean age of patients was 49 ± 10 years (81% women; 81% Caucasian). No significant differences in disease duration were found between patients with RA and DM, with a mean of 9.7 ± 8.7 years. Patients with DM had a significantly increased prevalence of HTN, HL, CAD, MI, and CVA when compared with the other two groups. No significant differences were noted in the prevalence of these conditions when comparing patients with RA and the GP. BMI, SBP, and triglycerides were significantly higher, and HDL and LDL were significantly lower, in patients with DM when compared with the other two groups (Table 2). Of note, 64.5% of patients with DM were taking statins, compared with 9.2% of patients with RA and 15.1% of the GP (*P *< 0.0001; Table 3). Patients with RA had a significantly lower SBP and significantly higher HDL compared with the GP.

**Table 1:** Baseline demographic characteristics of each cohort

*N = *251/group		Group A (RA)	Group B (DM)	Group C (GP)	P; All three	P; A versus C
Age	Years (SD)	48.5 (10.0)	49.1 (9.8)	49.0 (9.3)	0.8	

Gender	Male	47 (19%)	47 (19%)	47 (19%)	1	
	Female	204 (81%)	204 (81%)	204 (81%)		

Race	Caucasian	203 (81%)	203 (81%)	203 (81%)		
	African-American	27 (11%)	27 (11%)	27 (11%)		
	Asian	17 (7%)	17 (7%)	17 (7%)		
	Other	4 (< 2%)	4 (< 2%)	4 (< 2%)		

Disease duration	Years (SD)	10.0 (8.4)	9.3 (9.0)	N/A	0.08	N/A

Personal history	HTN	71 (28%)	130 (52%)	71 (28%)	< 0.0001	1
	HL	55 (22%)	114 (45%)	48 (19%)	< 0.0001	0.5
	CAD	5 (2%)	27 (11%)	6 (2%)	< 0.0001	1
	MI	5 (2%)	12 (5%)	2 (1%)	.01	0.4
	CVA	4 (2%)	14 (6%)	4 (2%)	.009	1

Family history	CAD	74 (30%)	95 (38%)	62 (25%)	0.005	0.3
	MI	30 (12%)	49 (20%)	28 (11%)	0.012	0.9
	CVA	35 (14%)	25 (10%)	25 (10%)	0.3	0.2

CRP		0.9	N/A	N/A		

Hemoglobin A1c		N/A	7.5	N/A		

CAD, coronary artery disease; CRP, C-reactive protein; CVA, cerebrovascular accident; DM, diabetes mellitus; GP, general population; HL, hyperlipidemia; HTN, hypertension; MI, myocardial infarction; N/A, not applicable; RA, rheumatoid arthritis.

**Table 2:** Baseline clinical characteristics of each cohort

*N = *251/group		Group A (RA)	Group B (DM)	Group C (GP)	*P*, All three	*P*, A versus C
Exercise status	No data	165 (66%)	145 (58%)	154 (61%)	< 0.0001	< 0.0001
	Never	30 (12%)	46 (18%)	29 (12%)		
	Infrequently 1-2×/week	15 (6%)	11 (4%)	9 (4%)		
	Moderately 3-5×/week	31 (12%)	18 (7%)	15 (6%)		
	Regularly > 5×/week	10 (4%)	31 (12%)	43 (17%)		

BMI		28.5 (7.5)	34.9 (8.9)	28.8 (7.9)	< 0.0001	0.4

Smoking status	Never	167 (67%)	155 (62%)	151 (60%)	0.2	0.7
	Past	48 (19%)	48 (19%)	44 (18%)		
	Current	36 (14%)	48 (19%)	56 (22%)		

SBP		121.8 (13.0)	130.5 (13.0)	125.0 (14.1)	< 0.0001	0.02

DBP		73.3 (7.6)	72.9 (8.6)	73.2 (8.0)	0.6	0.8

Number BP measurements		6.5 (5.8)	5.8 (2.9)	4.3 (3.0)	< 0.0001	< 0.0001

HDL		58.3 (17.1)	46.8 (12.9)	54.3 (15.9)	< 0.0001	0.01

LDL		113.7 (32.4)	99.5 (37.1)	111.4 (29.1)	< 0.0001	0.5

Triglycerides		116.9 (64.1)	192.8 (178.9)	130.6 (79.4)	< 0.0001	0.1

Fasting glucose	Mean (std)	90.3 (9.3)	165.5 (82.3)	93.6 (10.7)	< 0.0001	0.003
	*N*	206	166	175		

BMI, body mass index; BP, blood pressure; DBP, diastolic blood pressure; DM, diabetes mellitus; GP, general population; HDL, high-density lipoprotein; LDL, low-density lipoprotein; RA, rheumatoid arthritis; SBP, systolic blood pressure.

**Table 3:** Baseline intake of medications in each cohort

*N = *251/group		Group A (RA)	Group B (DM)	Group C (GP)	P, all three	P, A versus C
Medications						

RA	MTX	126 (50%)	N/A	N/A	--	--
	HCQ	92 (37%)	N/A	N/A	--	--
	PDN	86 (34%)	N/A	N/A	--	--
	TNF	96 (38%)	N/A	N/A	--	--

DM	Insulin	N/A	100 (40%)	N/A	--	--
	Metformin	N/A	132 (53%)	N/A	--	--

NSAIDs		74 (29%)	43 (17%)	51 (20%)	0.003	0.02

Anti-HTN		66 (26%)	180 (72%)	86 (34%)	< 0.0001	0.052

Statin		23 (9%)	162 (65%)	38 (15%)	< 0.0001	0.055

ASA	81 mg	22 (9%)	93 (37%)	33 (13%)	< 0.0001	0.12
	> 81 mg	5 (2%)	16 (6%)	6 (2%)	0.01	0.76

Anti-HTN, antihypertensives; ASA, aspirin; DM, diabetes mellitus; GP, general population; HCQ, hydroxychloroquine; LEF, leflunomide; MTX, methotrexate; N/A, not applicable; NSAIDs, nonsteroidal antiinflammatory drugs; PDN, prednisone; RA, rheumatoid arthritis; TNF, anti-TNF agents.

### Assessment of CRF identification and management in RA patients by rheumatologists compared with PCPs

**Figure 1:**
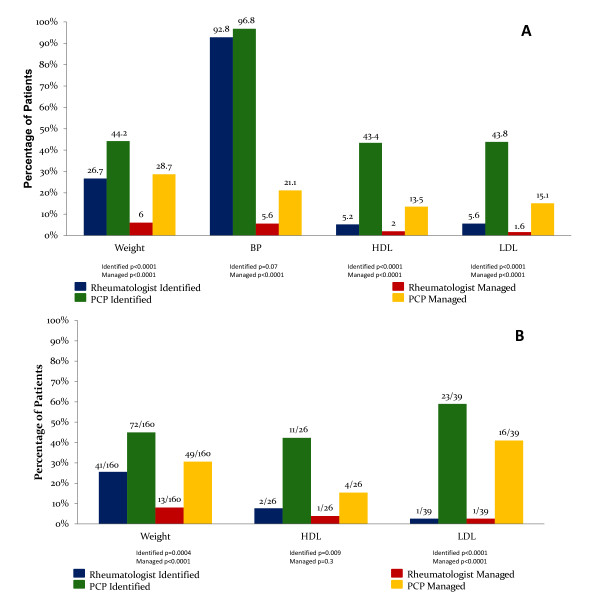
**CRF identification and management in patients with RA**. **(A) **CRF identification and management by rheumatologists compared with PCPs in RA patients. Results represent the percentage of RA patients whose CRFs were identified or managed by rheumatologists, compared with primary care physicians; the numeric value above each bar in the figure represents this percentage. **(B) **Suboptimal CRF identification and management by rheumatologists compared with PCPs in RA patients. All patients included in this subgroup analysis had RA and an abnormal value for a given risk factor. Results represent the percentage of RA patients whose abnormal CRFs were identified and/or managed by rheumatologists compared with primary care physicians. The fraction above each bar in the graph represents the number of patients whose abnormal CRF was identified or managed, divided by the total number of patients in the group with an abnormal CRF. BP, blood pressure; HDL, high-density lipoprotein; LDL, low-density lipoprotein; PCP, primary care physician.

When compared with rheumatologists, PCPs identified smoking status, weight, lipids, and FBG significantly more frequently in RA patients (Figure 1). Furthermore, compared with rheumatologists, PCPs managed weight, BP, lipids, and FBG significantly more frequently in RA patients. Weight was managed in 28.7% of RA patients by PCPs, almost 5 times as frequently when compared with rheumatologists. Similarly, LDL in RA patients was managed by PCPs almost 9 times as frequently as rheumatologists (Figure 1A).

The subgroup analysis revealed that, in RA patients with abnormal CRF values, PCPs identified smoking status, weight, systolic BP, HDL, LDL, triglycerides, and FBG significantly more frequently, when compared with rheumatologists (Figure 1B). Further, in RA patients with abnormal CRF values, smoking, weight, BP, LDL, and triglycerides were managed significantly more frequently by PCPs when compared with rheumatologists. In RA patients with elevated LDL, 41.0% were managed by PCPs, compared with 2.6% by rheumatologists (*P *< 0.0001).

An additional subgroup analysis was performed to determine whether an elevated CRP level, defined as ≥ 0.7 mg/dl, was associated with increased CRF identification and/or management in RA patients, by rheumatologists or PCPs. Neither rheumatologists nor PCPs identified CRFs any differently in RA patients with normal versus elevated CRP levels. However, rheumatologists managed BP and BMI significantly more frequently in those with an elevated CRP level as compared with those with a normal CRP level (12.2% versus 2.7%, *P *= 0.007 for BP, and 11.0% versus 3.3%, *P *= 0.04 for BMI). PCPs managed BP significantly more frequently in RA patients with an elevated compared with normal CRP level (34.2% versus 15.9%; *P *= 0.002).

### CRF identification and management by PCPs in patients with RA, DM, and of the GP

PCPs identified weight, lipids, and FBG significantly more frequently in patients with DM, when compared with those with RA or with the GP. In contrast, smoking status was identified significantly more frequently in the GP when compared with the other two groups (Table 4). In addition, PCPs identified weight, BP, and lipids significantly more frequently in the GP when compared with RA patients.

**Table 4:** CRF identification and management by rheumatologists in RA; PCPs in RA, DM, and the GP

		Group A (RA) *Rh*	Group A (RA) *PCP*	Group B (DM)	Group C (GP)	*P*, Group A (Rh versus PCP)	*P*, all three (PCP)	*P*, A versus C (PCP)
Smoking	ID	52 (21%)	167 (67%)	161 (64%)	188 (75%)	< 0.0001	0.02	0.0496
	MA	32 (13%)	25 (10%)	28 (11%)	39 (16%)	0.4	0.1	0.08

Weight	ID	67 (27%)	111 (44%)	191 (76%)	136 (54%)	< 0.0001	< 0.0001	0.03
	MA	15 (6%)	72 (29%)	159 (63%)	99 (39%)	< 0.0001	< 0.0001	0.01

BP	ID	233 (93%)	243 (97%)	250 (99.6%)	250 (99.6%)	0.07		0.04
	MA	14 (6%)	53 (21%)	169 (67%)	80 (32%)	< 0.0001	< 0.0001	0.008

HDL	ID	13 (5%)	109 (43%)	190 (76%)	144 (57%)	< 0.0001	< 0.0001	0.002
	MA	5 (2%)	34 (14%)	137 (55%)	70 (28%)	< 0.0001	< 0.0001	0.0001

LDL	ID	14 (6%)	110 (44%)	197(79%)	146 (58%)	< 0.0001	< 0.0001	0.004
	MA	4 (2%)	38 (15%)	160 (64%)	78 (31%)	< 0.0001	< 0.0001	< 0.0001

TG	ID	14 (6%)	109 (43%)	190 (76%)	141 (56%)	< 0.0001	< 0.0001	0.006
	MA	5 (2%)	36 (14%)	142 (57%)	70 (28%)	< 0.0001	< 0.0001	0.0003

FBG	ID	3 (1%)	58 (23%)	237 (94%)	68 (27%)	< 0.0001	< 0.0001	0.4
	MA	0	7 (3%)	232 (92%)	19 (8%)	0.02	< 0.0001	0.03

Percentages are calculated as the number of patients with an identified or managed risk factor divided by the total number of patients within the group. BP, blood pressure; CRF, cardiovascular risk factor; DM, diabetes mellitus; FBG, fasting blood glucose; GP, general population; HDL, high-density lipoprotein; ID, identified; LD, low-density lipoprotein; MA, managed; PCP, primary care physician; RA, rheumatoid arthritis; Rh, rheumatologist; TG, triglyceride.

Weight, BP, lipids, and FBG were managed significantly more frequently in DM patients, when compared with the other two groups. PCPs managed weight, BP, lipids, and FBG significantly more frequently in the GP, as compared with patients with RA.

**Figure 2:**
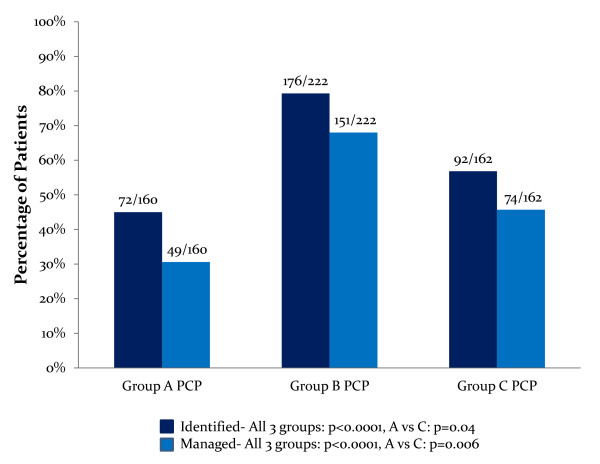
**Abnormal body mass index (BMI) identification and management by PCPs in rheumatoid arthritis (RA), diabetes mellitus (DM), andGP patients**. All patients included in this subgroup analysis had abnormal BMIs. Results represent the percentage of patients in each cohort whose abnormal BMI was identified and/or managed by their PCPs. The fraction above each bar in the graph represents the number of patients whose abnormal BMI was identified or managed divided by the total number of patients with an abnormal BMI in each group. PCP, primary care physician; Group A, RA patients; Group B, DM patients; Group C, general population; BMI, body mass index.

A subgroup analysis, evaluating patients with abnormal values for each risk factor, found that PCPs identified weight, lipids, and FBG and managed weight, BP, lipids, and FBG significantly more frequently in DM, when compared with RA or GP patients (Table 5). In this subset of patients, PCPs identified and managed abnormal weight significantly more frequently in patients of the GP when compared with patients with RA. In patients with elevated BMI, PCPs managed weight in 68.0% of DM patients, 45.7% of GP patients, and 30.6% of RA patients (*P <*0.0001 in all groups and *P *= 0.006 in GP versus RA groups; Figure 2).

**Table 5:** Subgroup analysis: identification and management of abnormal CRFs

		Group A (RA) Rh	Group A (RA) PCP	Group B (DM)	Group C (GP)	P, Group A (Rh versus PCP)	P, all three (PCP)	P, A versus C (PCP)
Smoking	Abnl (*n*)	36	36	48	56			
	ID	6 (17%)	29 (81%)	40 (83%)	49 (88%)	< 0.0001	0.7	0.4
	MA	5 (14%)	20 (56%)	25 (52%)	34 (61%)	0.0004	0.7	0.7

Weight	Abnl (*n*)	160	160	222	162			
	ID	41 (26%)	72 (45%)	176 (79%)	92 (57%)	0.0004	< 0.0001	0.04
	MA	13 (8%)	49 (31%)	151 (68%)	74 (46%)	< 0.0001	< 0.0001	0.006

SBP	Abnl (*n*)	132	132	199	150			
	ID	119 (90%)	131 (99%)	198 (99.9%)	149 (99%)	0.001	0.9	1
	MA	12 (9%)	44 (33%)	146 (73%)	64 (43%)	< 0.0001	< 0.0001	0.1

DBP	Abnl (*n*)	45	45	45	52			
	ID	43 (96%)	45	45	52	0.5	1	1
	MA	8 (18%)	18 (40%)	35 (78%)	30 (58%)	0.04	< 0.001	0.1

HDL	Abnl (*n*)	26	26	75	33			
	ID	2 (8%)	11 (42%)	56 (75%)	22 (67%)	0.009	< 0.01	0.07
	MA	1 (4%)	4 (15%)	38 (51%)	13 (39%)	0.3	< 0.0001	0.08

LDL	Abnl (*n*)	39	39	102	33			
	ID	1 (3%)	23 (59%)	85 (83%)	26 (79%)	< 0.0001	0.009	0.08
	MA	1 (3%)	16 (41%)	76 (75%)	21 (64%)	< 0.0001	0.0009	0.06

TG	Abnl (*n*)	53	53	130	54			
	ID	6 (11%)	31 (59%)	110 (85%)	36 (67%)	< 0.0001	< 0.0003	0.4
	MA	3 (6%)	17 (32%)	86 (66%)	20 (37%)	0.0009	< 0.0001	0.7

FBG	Abnl (*n*)	26	26	141	36			
	ID	0	12 (46%)	135 (96%)	16 (44%)	< 0.0001	< 0.0001	1
	MA	0	2 (8%)	132 (94%)	8 (22%)	0.5	< 0.0001	0.2

All patients included in this subgroup analysis had an abnormal value for a given risk factor. Percentages are calculated as the number of patients with an identified or managed risk factor divided by the number of patients with abnormal values for the given risk factor. Abnl, abnormal; DBP, diastolic blood pressure; DM, diabetes mellitus; FBG, fasting blood glucose; GP, general population; HDL, high-density lipoprotein; ID, identified; LDL, low-density lipoprotein; MA, managed; RA, rheumatoid arthritis; Rh, rheumatologist; SBP, systolic blood pressure; TG, triglyceride.

**Figure 3:**
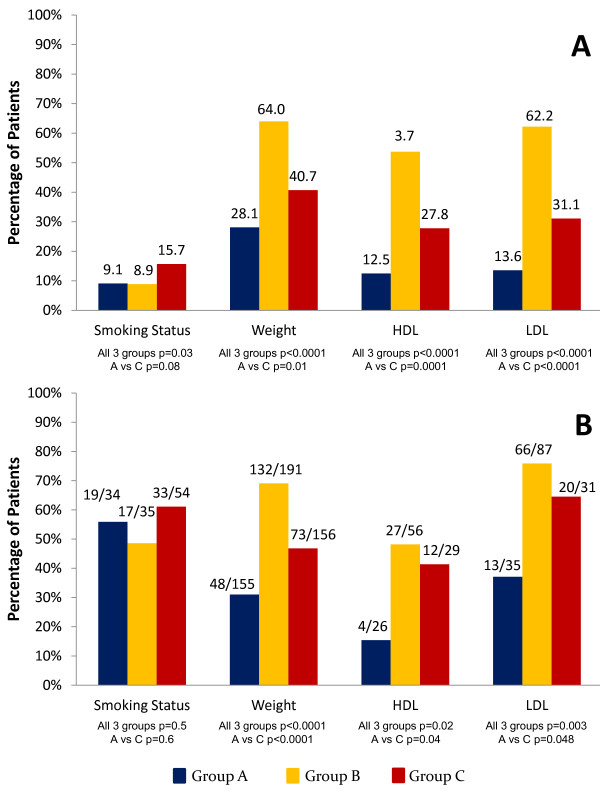
**CRF management by PCPs in RA, DM, and GP patients without a CVD history**. **(A) **CRF management by PCPs in RA, DM, and GP patients without CVD, as defined by a history of CAD, MI, and/or CVA. Results represent the percentage of patients whose CRFs were managed by PCPs; the numeric value above each bar in the figure represents this percentage. **(B) **Suboptimal CRF management by PCPs in RA, DM, and GP patients without a history of CVD. All patients included in this subgroup analysis had an abnormal value for a given risk factor and no CVD history. Results represent the percentage of patients whose abnormal CRFs were managed by PCPs. The fraction above each bar in the graph represents the number of patients whose abnormal CRF was managed, divided by the total number of patients with an abnormal CRF in the group. PCP, primary care physician; Group A, RA patients; Group B, DM patients; Group C, general population; BP, blood pressure; HDL, high-density lipoprotein; LDL, low-density lipoprotein.

An additional subgroup analysis excluding all patients with a history of CVD, defined as CAD, MI, and/or CVA, found that PCPs identified and managed smoking status, weight, BP, HDL, LDL, and triglycerides significantly more frequently in DM, when compared with RA and with GP patients (all *P *values < 0.05; Figure 3A). In addition, weight, BP, HDL, LDL, and triglycerides were identified and managed significantly more frequently in the GP patients, when compared with RA patients (all *P *values < 0.05). When assessing patients without a history of CVD and with abnormal values for each CRF, PCPs managed weight, BP, HDL, LDL, and triglycerides significantly more frequently in patients with DM, when compared with RA or the GP (all *P *< 0.05; Figure 3B). In these same patients, PCPs managed weight, LDL, and HDL significantly more frequently in GP as compared with RA patients (all *P *< 0.05).

Overall, rheumatologists identified and managed CRFs significantly less frequently in RA patients, when compared with PCPs. Abnormal BMI, BP, and lipid profiles were most frequently identified and managed by PCPs in patients with DM, compared with patients with RA. Abnormal BMI was more frequently identified and managed by PCPs in the GP, when compared with patients with RA.

## Discussion

In this cohort study performed at a tertiary-care center in the United States, we found that rheumatologists identify and manage CRFs in RA patients significantly less frequently than PCPs. This is in agreement with another study that reported that RA patients are better screened for CRFs when also followed up by a PCP rather than only by a rheumatologist. In particular, that study reported that RA patients who were followed up only by a rheumatologist, underwent lipid screening 22% of the time versus 43% to 51% when PCPs were also involved [15]. These results indicate the need for improved collaboration between PCPs and rheumatologists to screen RA patients for primary CRF prevention.

In addition, our study found that abnormal BMI, BP, and lipid profiles are most frequently identified and managed by PCPs in patients with DM than in those with RA. More surprising is that abnormal BMI is more frequently identified and managed by PCPs in the GP when compared with patients with RA. This indicates that, although patients with RA have a well-established increased CV risk, equivalent to the risk observed in DM, traditional CRFs in RA are not adequately identified and managed, even to a degree comparable with that of the GP [21]. This underrecognition and underassessment of CV risk in RA patients is a finding further discussed in a recently published study that involved sending questionnaires to PCPs and asking them about their awareness of and clinical practices regarding CV risk and RA [22]. In that study, only 32% of PCPs identified RA as an independent risk factor for CVD, and 15% of PCPs assessed RA patients for primary prevention of CV risks. In addition, they discovered that PCPs who had received some type of education on increased CV risk in RA more frequently assessed CV risk. However, of those PCPs, only 40% performed primary prevention risk assessment. These findings highlight the suboptimal awareness of CVD in RA among primary care providers, and further support our results.

Perhaps if better established primary prevention guidelines existed regarding CRF management in RA, as proposed by EULAR, more active management of these CRFs by providers would be observed [13]. Current models to predict the risk of CVD for the general population, such as the general Framingham and the Reynolds risk scores, underestimate CV risk in patients with RA, according to one study [23]. Indeed, the observed CVD risk in RA patients was twofold higher in women and 65% higher in men, when compared with that predicted by the Framingham risk score. These results emphasize the need for more accurate models to predict CV risk in RA. In addition, further studies should be pursued to determine the efficacy of the available EULAR recommendations, to better formulate and establish primary prevention guidelines regarding CRF management in RA.

The baseline characteristics in our study reveal no differences in the prevalence of some of the traditional CRFs and previous CV events between RA patients and the GP, in contrast to what may be expected, given the known increased CV risk in RA and prior findings in the published literature [6,7,24]. Also, RA patients in our study were found to have significantly lower SBP and significantly higher HDL when compared with the GP. This is also in contrast with other studies that reported lower HDL levels in RA compared with the GP [7,25]. These findings may be because the average age of patients in our study was 49 years, 8 years younger than the average age of patients in studies reporting higher prevalence of CAD, MI, and CVA, and/or lower HDL levels [3,25]. Furthermore, White *et al. *[26] found a statistically significant inverse relation between HDL and CRP levels. RA patients in our study were found to have relatively well-controlled disease, with overall low CRP levels. This could potentially explain the higher-than-expected HDL levels. Also, the mere quantification of HDL in RA may not reveal true CV risk, as these patients have higher prevalence of oxidized HDL, which loses its antiinflammatory properties and may become proatherogenic [27]. In addition, our study excluded patients with RA coexisting with DM to reduce confounding factors. This may have skewed our baseline characteristics, given that these patients with the highest risk were excluded.

Our study found that DM patients had significantly lower LDL levels compared with the other two groups, which may be because a significantly higher percentage of diabetes patients were taking a statin (*P *< 0.0001). As expected, DM patients had significantly higher BMI, SBP, and triglyceride measurements.

Limitations of our study include that rheumatologists may have not addressed certain CRFs in RA because they were aware that their patients were also being followed up by a PCP. It is possible that the same rheumatologist would have more actively identified and managed these CRFs if the patient were not seen by a PCP. Similarly, rheumatologists may not have addressed CRFs with RA patients if these patients were first examined by a PCP. It should be kept in mind that, although PCPs were shown to identify and manage CRFs better in RA compared with rheumatologists, they still did not adequately address their CRFs when compared with the GP. Therefore, even when patients are followed up by PCPs, rheumatologists should be vigilant about the detection and management of CV risk in RA patients.

A second limitation of our study is that the data were gathered retrospectively from medical charts. Hence, if the provider identified or managed a CRF but did not document it, the particular CRF would be considered as not addressed for the purposes of our study. We assume that this likely occurred equally in all three groups, so the relative discrepancies seen would still be present.

Our study has several strengths, including that we performed an extensive chart review of a large cohort of patients (*n *= 753). Although a prior study assessed the management of individual CRFs, to our knowledge, no other studies performed such a thorough assessment of the management of multiple CRFs in RA [15]. In addition, no other studies have simultaneously assessed CRF management in patients with RA when compared with those with DM, a more-established CAD risk equivalent.

Finally, our study accounted for the overall CVD history and for individual risk-factor abnormalities in the subgroup analyses. We found that, at baseline, there was about a fivefold increase in the prevalence of a history of CVD in DM as compared with RA or GP patients. Therefore, the higher frequency of CRF identification and management found in DM patients could potentially have been due to the increased prevalence of CVD in this group. However, when excluding patients with a history of CVD, we found that PCPs still identified and managed certain CRFs most frequently in DM, followed by GP, and then RA patients. These findings persisted when looking at patients without CVD and with abnormal CRF values.

## Conclusions

In summary, we found that despite rheumatologists' awareness of increased CV risk in RA patients, they are significantly less likely to identify and manage CRFs when compared with PCPs. In addition, the management of CRFs by general practitioners in RA patients is suboptimal, when compared with strategies implemented in the GP and DM patients. We hypothesize that health care providers spend more time focusing on rheumatologic issues rather than seemingly less-pressing primary prevention issues. However, given the well-established increased CV risk associated with RA, both rheumatologists and PCPs should collaborate and more aggressively identify and manage CRFs in patients with RA to minimize their deleterious effects on morbidity and mortality.

## Abbreviations

CAD: coronary artery disease; CRF(s): cardiovascular risk factor(s); CV: cardiovascular; CVD: cardiovascular disease; DM: type 2 diabetes mellitus; GP: general population; HDL: high-density lipoprotein; HL: hyperlipidemia; HTN: hypertension; LDL: low-density lipoprotein; PCP: primary care physician; RA: rheumatoid arthritis.

## Competing interests

The authors declare that they have no competing interests.

## Authors' contributions

SD participated in the design of the study, collected the data, and wrote the manuscript. JM participated in the design of the study and performed the statistical analysis. MK conceived of the study, participated in its design and coordination, and helped to draft the manuscript. All authors read and approved the manuscript for publication.
